# Investigation of Additive-Manufactured Carbon Fiber-Reinforced Polyethylene Terephthalate Honeycomb for Application as Non-Pneumatic Tire Support Structure

**DOI:** 10.3390/polym16081091

**Published:** 2024-04-13

**Authors:** Siwen Wang, Pan He, Quanqiang Geng, Hui Huang, Lin Sang, Zaiqi Yao

**Affiliations:** 1 School of Mechanical Engineering, Dalian University of Technology, Dalian 116024, China; 2018dgclgcwsw@mail.dlut.edu.cn (S.W.); hepan_jy@163.com (P.H.); cat12345dogsz@163.com (Q.G.); 2 School of Control Science and Engineering, Dalian University of Technology, Dalian 116024, China; huihuang@dlut.edu.cn; 3 School of Materials Science and Engineering, Dalian University of Technology, Dalian 116024, China; 4 Zhejiang Key Laboratory of Automobile Safety Technology, Geely Automobile Research Institute (Ningbo) Co., Ltd., Ningbo 315336, China

**Keywords:** honeycomb structures, 3D printing, non-pneumatic tire, mechanical property, fiber-reinforced polymer composites

## Abstract

A non-pneumatic tire (NPT) overcomes the shortcomings of a traditional pneumatic tire such as wear, punctures and blowouts. In this respect, it shows great potential in improving driving safety, and has received great attention in recent years. In this paper, a carbon fiber-reinforced polyethylene terephthalate (PET/CF) honeycomb is proposed as a support structure for NPTs, which can be easily prepared using 3D printing technology. The experimental results showed that the PET/CF has high strength and modulus and provides excellent mechanical properties. Then, a finite element (FE) model was established to predict the compression performance of auxetic honeycombs. Good agreement was achieved between the experimental data and FE analysis. The influence of the cell parameters on the compressive performance of the support structure were further analyzed. Both the wall thickness and the vertically inclined angle could modulate the mechanical performance of the NPT. Finally, the application of vertical force is used to analyze the static load of the structure. The PET/CF honeycomb as the support structure of the NPT showed outstanding bearing capacity and stiffness in contrast with elastomer counterparts. Consequently, this study broadens the material selection for NPTs and proposes a strategy for manufacturing a prototype, which provides a reference for the design and development of non-pneumatic tires.

## 1. Introduction

The tire is an important part of an automobile and a highly engineered composite structure, which directly influences the automobile’s safety and comfortableness [1,2,3]. It is the only medium for connecting the vehicle’s body and the road surface. It not only carries the weight of the vehicle body, but also absorbs the energy inverted by the road through its own deformation. In recent years, research has attempted to replace pneumatic tires with non-pneumatic tires (NPTs) using elastic materials or deformable honeycomb structures [4,5].

The air-free tires represented by Michelin Tweel tires are mainly made of polyurethane material and are formed by pouring. In recent years, the thermoplastic polyurethane with super elasticity has gradually attracted attention, making it possible for the application of 3D printing technology in the preparation of air-free inflatable tires [6,7]. Various additives are used as enhancing materials for NPT support structures, in which carbon nanotubes have significant effects in enhancing the strength and wear resistance of the composites, but the preparation cost is high. Their application in inflatable tires is limited, and carbon fiber is a more economical alternative. In addition, the use of glass fiber for the NPT support structure has been verified [8].

An NPT has been increasingly proposed due to its low risk of puncture, tire blowout or other safety hazards. In particular, the inner deformable support body provides good buffering and damping advantages, which greatly affect the mechanical properties and stress concentration of NPT performance [8]. Researchers have utilized porous structures to fill the NPT’s deformable body part due to its lightweight, load-bearing capacity and excellent energy absorption property [9,10,11,12].

For the working condition of an NPT, the vertical load and the energy absorption are the main evaluation of performance indexes [13,14,15,16,17]. Therefore, the compression property of the honeycomb structure is one of the main evaluating methods used to assess the structural performance of NPT support structures.

Regarding current developments, two commercial NPTs, including Tweel and AirFree Concept (AC), have successively been proposed. Thus, the vertical stiffness among the honeycomb, AC and Tweel was numerically analyzed and compared with the same spoke thickness [18,19,20]. Moreover, Rugsaj et al. proposed an interconnected mesh-spoke NPT model and compared it with the honeycomb and commercial NPT structures [21]. Zang et al. investigated the mechanical characteristics of an NPT with a rhombus structure under pavement conditions, which provided guidance for the structural optimization of NPT [22]. However, the abovementioned studies were mainly focused on a simulated analysis, and experimental tests of the deformable structure or prototype sample have seldom been investigated.

Due to the rapid development of polymer materials and additive manufacturing techniques, NPT support structures with new geometric shapes have been widely investigated [23,24,25,26]. Three-dimensional printing provides the possibility to realize the complicated honeycomb structures, which could be used to investigate the mechanical behavior of the designed geometric model in an experimental test [27,28,29]. The in-plane compressive or impact crashworthiness performance of 3D-printed samples are compressively studied and assessed [30,31,32]. The combination of an experimental test and numerical analysis provides a more accurate assessment for the 3D-printed honeycomb structures. Auxetic structures with a negative Poisson’s ratio show great advantages in terms of good energy absorption, high compressibility and improved damping performance. In previous work, 3D-printed re-entrant structures were fabricated using different polymeric materials, including pure resin and fiber-reinforced composites. In particular, the fiber-reinforced composite re-entrant samples possessed excellent load-carrying capacity and energy absorption capacity [33,34,35]. Moreover, these 3D-printed honeycombs exhibited typical layer-by-layer folding deformation behavior when they suffered a large compressive displacement, which significantly improved the structural flexibility [36,37]. Accordingly, the auxetic structure with a re-entrant cellular unit might be an alternative for the NPT deformable body.

In this paper, a concave honeycomb made from PET/CF material is presented. The 1:10 scaling model is thoroughly tested, and the finite element model is verified by benchmarking the deformation mode and mechanical response. Geometric parameters are used to analyze the structure, including the force absorption behavior under a large deformation and the natural frequency of free modes. Finally, the vertical stiffness of the structure is analyzed.

## 2. Materials and Experimental Methods

### 2.1. Materials

Commercial carbon fiber-reinforced polyethylene terephthalate (PET/CF) was purchased from FusRock Co., Ltd. (Suzhou, China). The raw filament had a density of 1.30 g/cm^3^, a melting temperature of 251 °C and a heat distortion temperature (HDT) of 112 °C (1.80 MPa). The content of carbon fiber was reported at 15 wt%.

### 2.2. Three-Dimentional Printing of PET/CF Samples 

The 3D printing of PET/CF filament was performed via a fused deposition modeling (FDM) 3D printer. The processing parameters were set as follows. The nozzle diameter was 0.4 mm, the nozzle temperature was 280 °C, the printing speed was 25 mm/min and the layer thickness was 0.15 mm. 

### 2.3. Design of the Testing Specimens and Honeycomb Structures 

**Figure 1:**
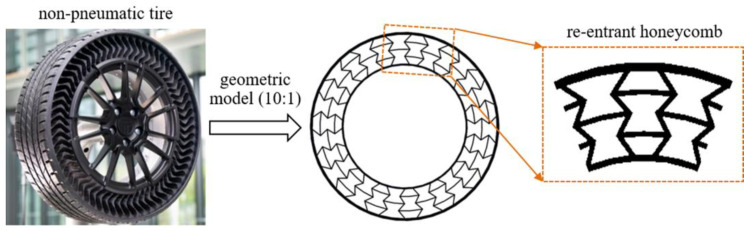
Structure of NPT with re-entrant deformable structure.

The testing specimens for tensile and flexural tests were designed using UG, exported into STL files and 3D-printed. Proportionally reduced model for non-pneumatic tire was designed and is illustrated in Figure 1. In our work, the deformable body part was filled with re-entrant honeycomb, which is illustrated in Figure 1. For NPT model, the outside diameter was 6400 mm and the tread width was 215 mm. The outer tire thickness was 10 mm and the inside hub connection thickness was 7mm. To realize the designed NPT deformable body, the 3D-printed model was proportionally scaled down in an equal ration of 10:1. 

### 2.4. Characterization 

Differential scanning calorimeter (DSC). The thermal parameters of PET/CF were evaluated using DSC. The samples weighed 5~8 mg and were sealed in an aluminum pan and conducted in nitrogen atmosphere. The sample was firstly heated to 280 °C to erase the thermal history, then cooled down to 25 °C and re-heated to 280 °C for the second heating. The cooling and heating rates were both set at 10 °C/min. 

The rheological behavior was characterized using a rotary rheometer (Anton Paar, MCR102e, North Ryde, Australia). Samples were 3D-printed into parallel plate with a diameter of 25 mm to conduct dynamic frequency scanning. The angular frequency ranged from 0.1 to 100 rad/s, and the test temperature was set at 280 °C.

Scanning electron microscopy (SEM). The fiber distribution in the PET matrix was observed using SEM (QUANTA 450, FEI, Gräfelfing, Germany). The filament was brittle fractured in liquid nitrogen and then sprayed with a thin gold layer. 

The tensile and flexural properties of 3D-printed PET/CF samples were tested by using a universal mechanical testing machine (TSE105D, Shenzhen Wance Testing Machine, Shenzhen, China) according to ISO 527 [38] and ISO 178 standards [39], respectively. The crosshead speed was 2 mm/min and was conducted at ambient temperature. The strength and modulus were obtained through the experimental data. 

The compressive test of the deformable structures was conducted principally in ISO 604 standard [40]. The ring-shaped re-entrant samples were placed between two rigid flat steel plates with a constant strain rate of 2 mm/min. The compressive force and displacement data from the universal machine were recorded. The total energy absorption (EA) was calculated according to our previous work [41]. 

## 3. Experimental Results and Discussion 

### 3.1. Thermal, Rheological and Mechanical Properties of PET/CF Filaments 

**Figure 2:**
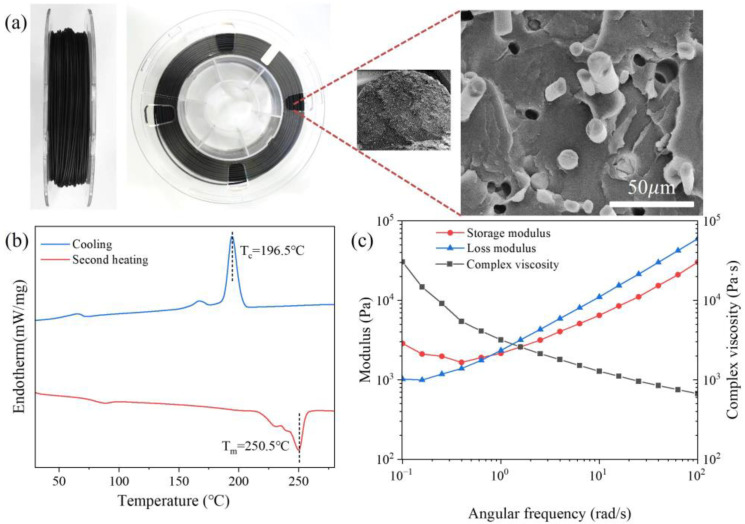
(**a**) The cross-sectional microstructure, (**b**) the first cooling and the second heating DSC curve and (**c**) the rheological behavior of PET/CF filament.

It can be seen that the short carbon fibers were evenly distributed in the PET/CF composite filament (Figure 2a), which provided sufficient strength and stiffness. Prior to 3D printing, the thermal and rheological behavior of PET/CF composite filament were studied (Figure 2b,c). As shown in Figure 2b, PET is a semi-crystalline polymer with an obvious crystallization temperature (T*_c_*) of 196.5 °C and a crystallization temperature (T*_m_*) of 250.5 °C. Therefore, the nozzle temperature was controlled from 280 to 300 °C, which was beneficial for good fluidity and smooth extrusion. 

From the rheological curves, both the storage modulus (G′) and loss modulus (G″) grew with the increase in the angular frequency. Under the lower angular frequency, the G′ values were higher than the G″ values, which then interacted. When reaching a higher frequency, the G″ values exceeded the G′ values, suggesting a transition from elasticity to viscosity for PET/CF composites. Meanwhile, the complex viscosity gradually decreased as the angular frequency increased, which indicates a typical shear thinning behavior.

**Figure 3:**
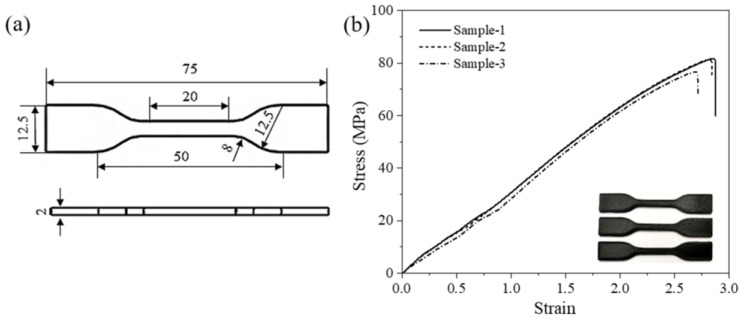
(**a**) The dimension of tensile specimen (unit: mm) and (**b**) tensile stress–strain curves of 3D-printed PET/CF.

**Figure 4:**
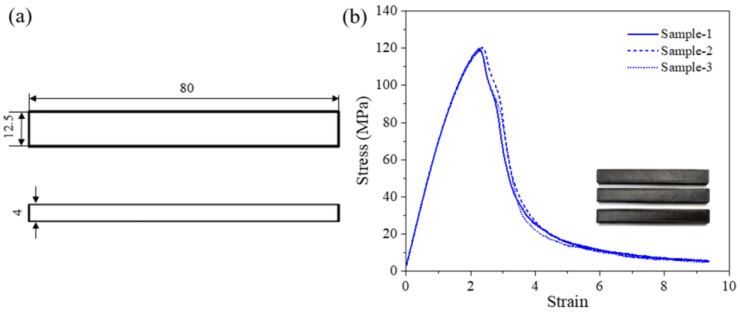
(**a**) The dimension of flexural specimen (unit: mm) and (**b**) flexural stress–strain curves of 3D-printed PET/CF.

The mechanical properties of the 3D-printed PET/CF specimens were evaluated and the parameters were obtained. The tensile properties of 3D-printed PET/CF specimens are presented in Figure 3. The results show that the stress–strain curves between parallel samples had high repeatability. The tensile strength and Young’s modulus were 80.5 ± 3.2 MPa and 3442.3 ± 44.2 MPa, respectively. On the other hand, the flexural specimens achieved a strength of approximately 119.4 ± 1.0 MPa and a modulus of 6924.7 ± 179.2 MPa (Figure 4). The statistical data are summarized in Table 1. Both the tensile and the flexural results demonstrated that sufficient strength and stiffness characteristics were obtained from the PET/CF filament. 

### 3.2. Three-Dimensional Printing of Deformable Re-Entrant Body and Compressive Performance 

**Figure 5:**
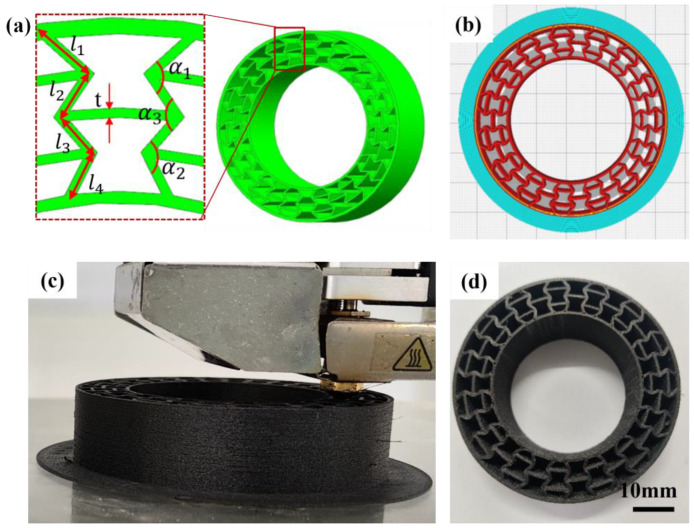
(**a**) The geometric model, (**b**) sliced model, (**c**) 3D-printing process and (**d**) the fabricated parts using PET/CF filament.

Based on the above analysis, the deformable body of an NPT with re-entrant honeycomb structures was 3D-printed. As shown in Figure 5, the scaled-down NPT model was designed using UG10.0 software. In detail, the outer thickness (*t*_1_) of the re-entrant structure was 1 mm and the inner thickness (*t*_2_) was 0.7 mm, whereas the middle layer thickness (*t*) was 0.6 mm. The thickness of the inclined struts was as follows: *l*_1_ = 3.2 mm, *l*_2_ = 3.1 mm, *l*_3_ = 2.9 mm and *l*_4_ = 2.8 mm. The angles between the inclined strut and horizontal direction were *α*_1_ = 98°, *α*_2_ = 112° and *α*_3_ = 105°. After the established model was sliced, the NPT deformable structure was successfully printed with a fine surface and interlayer quality. The good printability of PET/CF shows its great potential in manufacturing complicated parts. 

**Figure 6:**
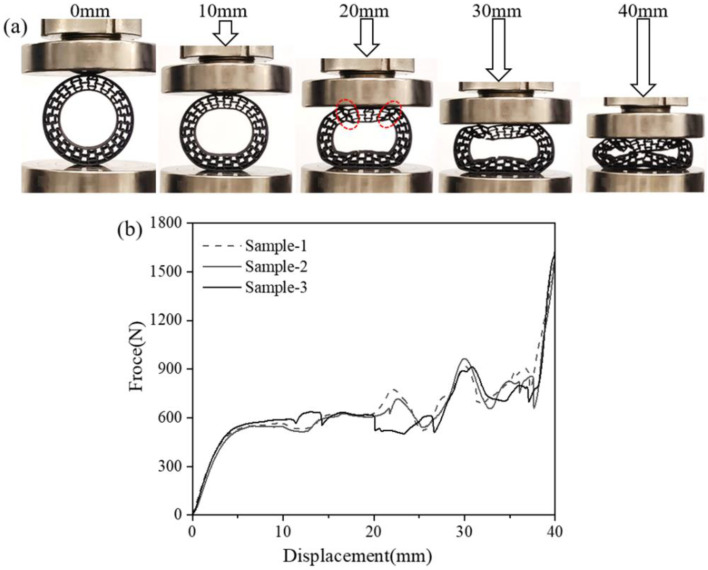
(**a**) The compressive process and (**b**) force–displacement curves of PET/CF deformable NPT part with re-entrant structures.

Then, the 3D-printed re-entrant structure was placed between the rigid flat and the compressive test was conducted. The compression test was similar to the research method of the basic honeycomb structure. The compressive test was similar to the working condition of the vertical loading for the NPT. As displayed in Figure 6a, the compression process accompanied by varying displacement was recorded using an advanced contactless charge-coupled device during the compression per one second. A large compressive displacement from 0 to 40 mm was conducted for the ring-shaped honeycombs. Cells that begin to become severely distorted are marked in red circles. It can be seen that the whole sample remained integrated and distorted during the whole compressive process, suggesting a sufficient toughness and stable state for the current structure. The corresponding curve is presented in Figure 6b. All the parallel samples showed high repeatability, which confirms the compressive behavior of PET/CF re-entrant structures. It was shown that the compressive stress–strain curves consisted of an initial elastic stage, a fluctuating plateau stage and a densification stage. The deformation process of the re-entrant honeycomb matched the curve’s variation pattern. 

When the displacement reached 10mm, the spherical shape changed to an oval shape without obvious distortion or damage, which is mainly attributed to the specific inward folding behavior of the re-entrant honeycomb. For the corresponding force–displacement curve (10 mm), a directly ascending and smooth plateau stage is observed. By increasing the displacement to 20 mm, a more obvious folding can be seen for the upper part of the sample, which carried and distributed the compression force. At this stage, no damage was detected for the compressed sample. As the compression continued, local fractures were detected in the upper and bottom part of the sample, suggesting a failure and collapsing failure mode. The fluctuation in the plateau stage with a displacement from 10 to 30 mm is attributed to the local buckling of the re-entrant sample. When the displacement reached 40 mm, an entire densification of the honeycomb was finally achieved, which is assigned to a sharply increasing force.

## 4. Numerical Modelling 

### 4.1. Validation of Simulation and Experimental Data of 3D Printing of Deformable PET/CF Re-Entrant Honeycomb 

**Figure 7:**
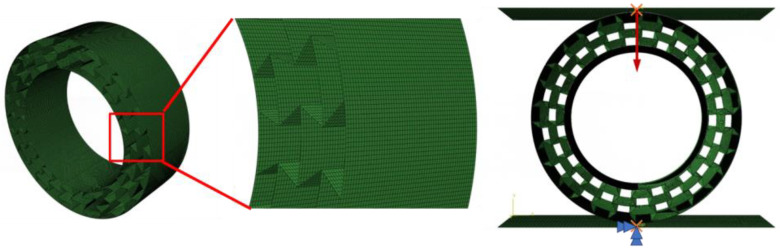
The FE model of ring-shaped re-entrant structure under compression load.

In this part, the finite element analysis (FEA) is performed to simulate and further analyze the in-plane compressive characteristic of the deformable PET/CF re-entrant honeycomb. A sufficient displacement load (70%) was applied to verify the complete compression behavior of the structure. Firstly, an FE model of the ring-shape re-entrant structure was constructed and verified with the experimental results. As displayed in Figure 7, an explicit version of ABAQUS 2021 was utilized; the target honeycomb adopted a first-order linear shell element (S4R) and was defined as a deformable body, whereas the upper and lower supporting platforms were defined as rigid plates. The neutral axis algorithm was chosen to ensure the uniformity of nodes, which were arranged along the surface. Considering the calculation efficiency, a mesh size of 0.4 mm is was for the following simulation analysis. The total number of elements was 136,080 and there were no error warnings for checking the mesh quality. For the supports, a two-dimensional flat plate simulation test machine was used to define the interaction for the assembly, with the contact field being all with self. A reference point was set and coupled with the pressure plate plane. The lower plate was fixed with six degrees of freedom and the upper plate was subjected to a displacement in the negative y-axis direction, as shown in Figure 7. Furthermore, the quality scaling function was used to improve the computational efficiency. 

**Figure 8:**
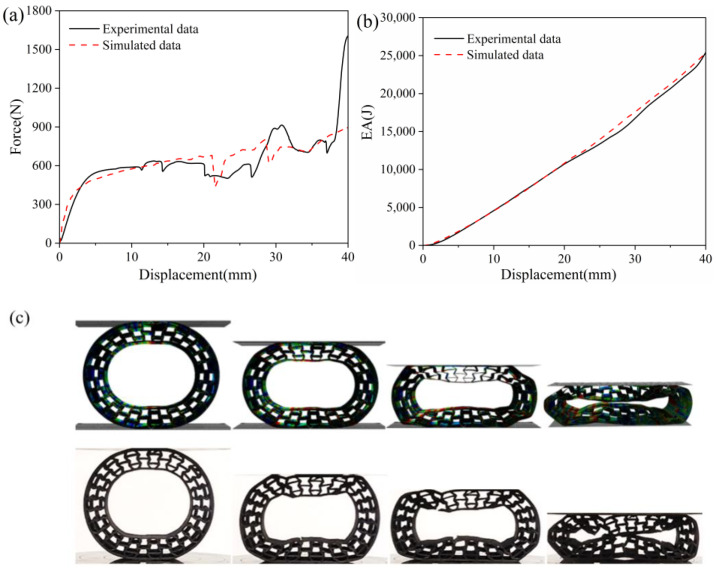
The verification between the experimental data with the simulated analysis, (**a**) the force–displacement and (**b**) the EA–displacement curves, and (**c**) the deformation behavior and von Mises distribution of PET/CF re-entrant samples.

Figure 8 shows the comparison between the experimental force–displacement curves and the EA–displacement curves with the numerical results. It can be seen that a good agreement was obtained for both the force–displacement and EA–displacement curves between the experimental data and simulation analysis, suggesting the accuracy and effectiveness of the numerical analysis models. In detail, the initial vertical compression load and the slope of the load–displacement curves were in good agreement, which is indicative of vertical load-bearing and stiffness. Furthermore, the integral area under the force–displacement is assigned to the absorbed energy, which was caused by the deformation of the re-entrant prototype samples during the load-bearing process. Therefore, the flexible deformation and high absorbed energy meant better buffering and damping performance [42].

Furthermore, the von Mises stress distribution for the PET/CF honeycomb structure during the compression process was also interpreted. The experimental records are also listed for the comparison. As displayed, no obvious stress concentration was shown in the FE models, suggesting an even stress distribution suffered by the proposed structures. Accordingly, the re-entrant samples exhibited good deformable behavior under low displacement, which is suitable as an NPT deformable body. It can be speculated that the NPT with re-entrant structures is more beneficial to distribute contact pressure.

### 4.2. Influence of Geometric Parameters on the Compressive Behavior of 3D-Printed PET Re-Entrant Samples 

**Figure 9:**
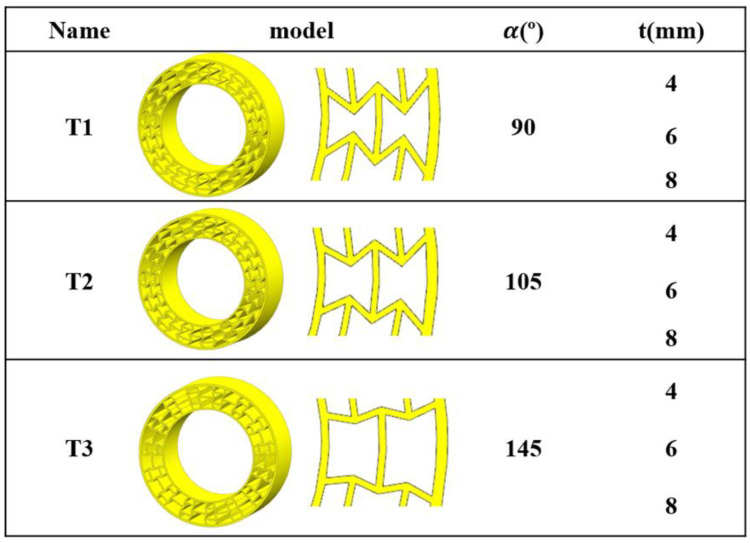
The variation of geometric parameters of PET/CF NPT deformable structures.

On the basis of the findings, the FE NPT deformable part models were established and were further employed to describe the impact of the geometric parameters on the compressive performance of the PET/CF NPT structures. The model as wis similar to that of re-entrant structure, and the vertical displacement was loaded to 300 mm; the force–displacement curves were simulated and exported. In detail, the geometric parameters, including angles of the vertically inclined strut and middle layer thickness, were selected and their variation is listed in Figure 9. The established models changed with the variation in the geometric parameter, which is coded as T1, T2 and T3. The wall thickness could be assigned in different shell thicknesses in the ABAQUS software.

**Figure 10:**
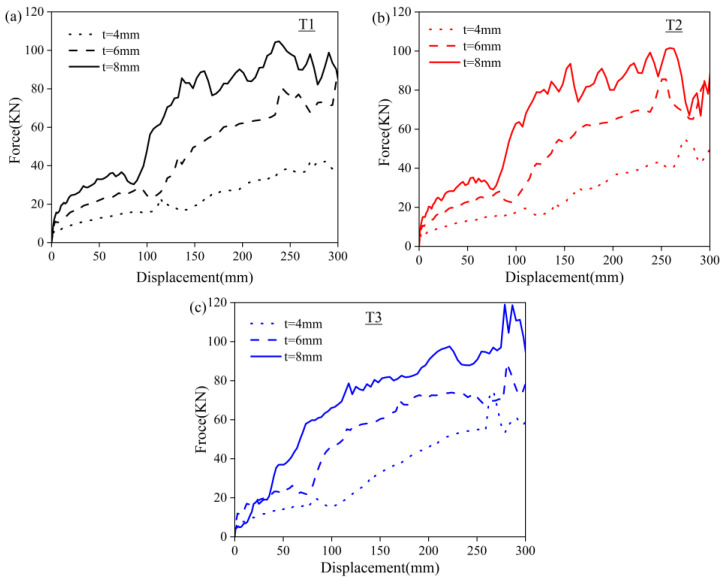
The prediction compressive force–displacement curves of PET/CF NPT deformable structures with different geometric parameters, (**a**) T1, (**b**) T2 and (**c**) T3 samples.

Based on the simulated models, the NPT structure with three different angles and cellular thicknesses were obtained. The simulations for different geometric parameters were conducted to study the mechanical response and energy absorption potential of structures during intact compression and the predicted curves are presented in Figure 10. It can be seen that the curve variation trend is similar to the scaled-down re-entrant prototype (as shown in Figure 6). With the increase in cell thickness, an increased bearing capacity was achieved. The curves can be divided into two ascending stages. The displacement of the first stage is mainly assigned to the stable elastic deformation of the re-entrant structures, whereas the second stage fluctuated when the displacement was above 150 mm. When the vertical load initially exerts force, the vertical displacement of the tire was small. The first peak came earlier with a larger angle, while the fluctuation became less intense. As the load increases, the support of the tire might lead to an excessive deformation.

**Figure 11:**
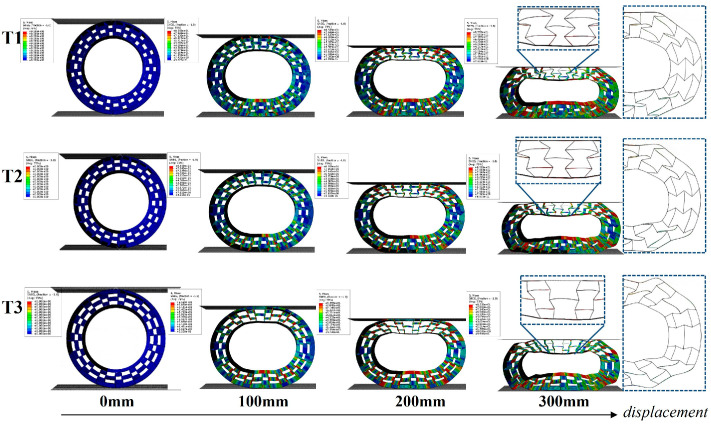
The predicted compressive force–displacement curves of PET/CF NPT deformable structures with different geometric parameters (T1, T2 and T3).

The representative simulated deformation process of NPT honeycombs (t = 6mm) for loading is shown in Figure 11. The deformation behavior with different angles was similar. Under a small displacement, the NPT honeycombs remained in a stable load-bearing state. With an increasing displacement, the upper part of the re-entrant model that contacted the flat plate did not greatly deform, but the ends of the cellular structure of the ellipse were squeezed and distorted (in the magnified region). Meanwhile, the bottom re-entrant part bulged and there was a stress concentration under a large displacement. 

**Figure 12:**
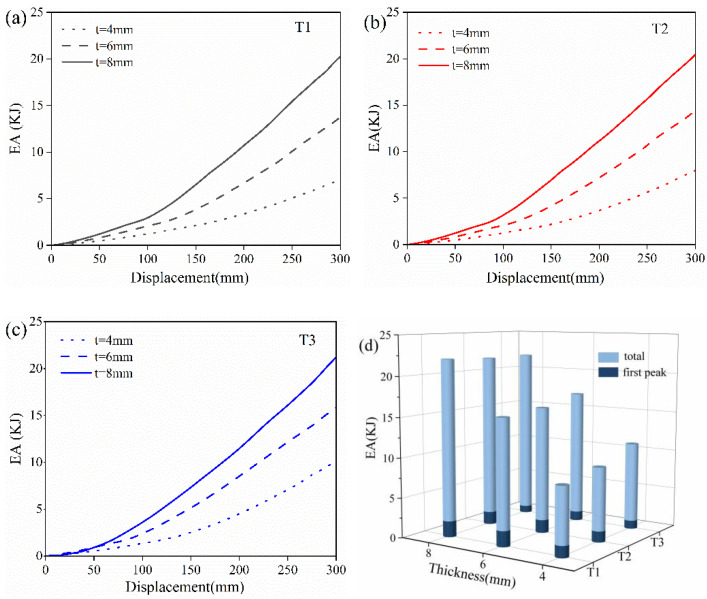
The simulated EA–displacement curves of (**a**) T1, (**b**) T2 and (**c**) T3 of NPTs with re-entrant structures and (**d**) EA histograms with different cell thicknesses.

The EA–displacement curves for deformable structures with different cell thickness were calculated and summarized in Figure 12. For the T1 structure with α = 90°, the total EA increased from 6.95 kN to 20.06 kN when the cell thickness rose from 4 mm to 8 mm. Similar trends were also observed for the T2 (α = 105°) and T3 (α = 145°) structures with varying thicknesses. On the other hand, larger total EA values were obtained when the angle increased under the same cell thickness. For instance, the total EA values of T1, T2 and T3 with 4 mm thickness were 6.95, 7.87 and 9.99 KJ, respectively. However, the EA of the first peak showed different patterns from the total EA values. The higher EA of the first peak more likely occurred with a smaller angle and thicker cells. Therefore, it is important to consider the working condition of NPTs when designing the deformable body. For good road conditions or small-load vehicles, the NPT suffers a low bearing load, and thus a honeycomb with α = 90° might be more suitable than those with α = 105° and α = 145°. On the contrary, once the load condition is worse or the vehicle is heavily loaded, an NPT with α = 145° might exhibit better compressive performance. 

### 4.3. Free Vibration Condition for NPT Deformable PET/CF Re-Entrant Structures 

**Figure 13:**
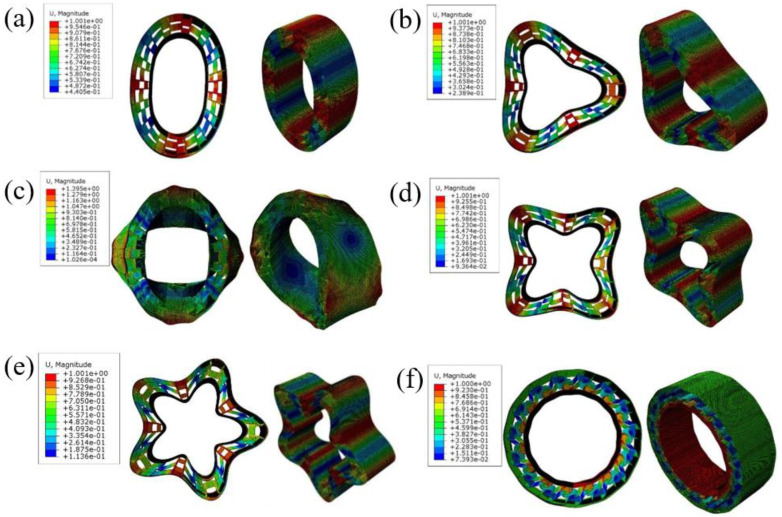
(**a**–**f**) show the 7–12 vibration modes of the NPT structure.

A modal analysis of the NPT structure is of great significance for controlling the wheel vibration as well as the resulting resonance and noise problems. Therefore, the modal analysis of the proposed re-entrant structures is reported in this section. The vibration modes of tires are generally divided into circumferential, transverse and radial. The representative [c,m] method is adopted to classify the modal vibration modes of NPT. Among them, c represents the number of sinusoidal fluctuations in the circumferential direction of the negative Poisson’s ratio microstructure pneumatic tire, while m represents the number of fluctuations in the radial direction of the tire. The frequency analysis in ABAQUS defaults to using the Lanczos solver, which can solve for a certain number of eigenvalues or define the frequency range to be solved. In this work we chose a linear vibration analysis, without applying boundary condition constraints, to extract the first 12 natural frequencies. The first six orders of free vibration comprised rigid body motion, in which the structure does not deform and the vibration frequency is 0. Figure 13 shows the 7–12 vibration modes of the NPT structure, including circumferential, transverse and radial modes. As the vibration frequency increases, the deformation of the supporting structure becomes more complex.

**Figure 14:**
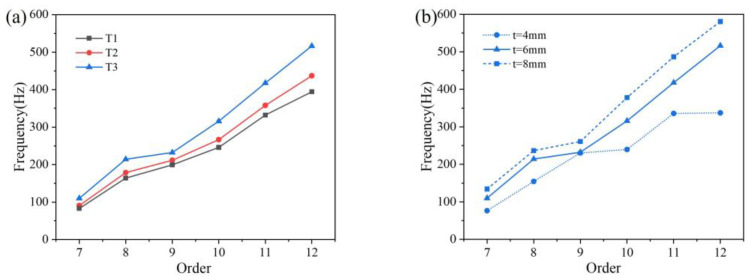
The natural frequency of NPT structure with (**a**) three different angles and (**b**) T2 with different thicknesses for representative example.

Figure 14a shows the natural frequencies of three types of tires with a wall thickness of 6 mm. As the internal angle of the microstructure increases, there is an increasing trend from the 7th natural frequency to the 12th natural frequency. Figure 14b shows the natural frequencies of T2 tires with different wall thicknesses. As the thickness increases, the magnitude of the natural frequencies increases. The effect of thickness variation on the 7–9 natural frequencies was relatively low, but it had a significant impact on the natural frequencies after the 10th order. Choosing appropriate parameters for the filling structure during design has a significant impact on reducing the low order natural frequency of pneumatic tires.

### 4.4. Analysis of Static Load of Non-Pneumatic Tire with Rim Support Structure

**Figure 15:**
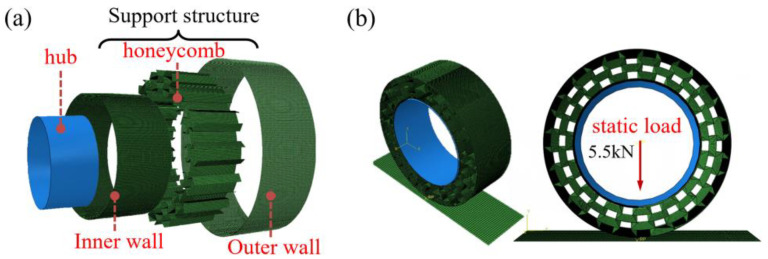
The finite element model of non-pneumatic tire: (**a**) structural composition and (**b**) boundary constraints and loading conditions.

In this section, the finite element model of support structure of NPT is reported, and the bearing capacity as well as the vertical stiffness are studied. As shown in Figure 15, a rigid wheel hub was connected to the support structure using a tie, and the ground was set as a rigid plate with frictional contact with the support structure. Assuming the vehicle was stationary, a vertical force of 5.5 kN was applied as the tire load. Deformation occurred at the contact point between the structure and the ground, and the Y-axis displacement of the central node was the output.

**Figure 16:**
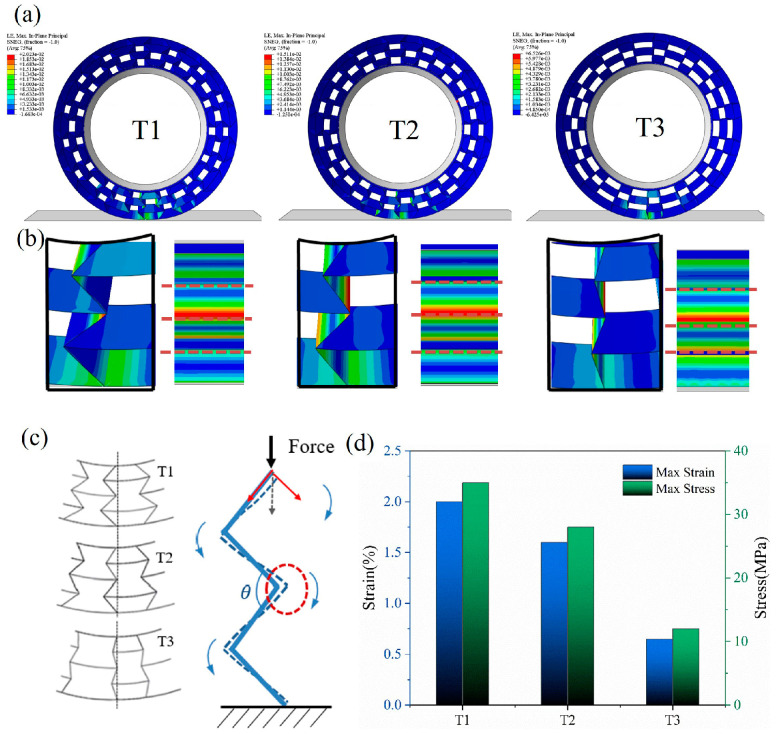
(**a**) Numerical analysis of support structure with a vertical load of 5.5 kN; (**b**) the stress distribution and the cell deformation; (**c**) diagram of stress and deformation of honeycomb wall; and (**d**) the maximum stress and strain with different cell angles.

Figure 16a shows the simulated results of the NPT support structure under a load of 5 kN, and the details of the stress distribution are shown in Figure 16b. In the plane which the cell wall was located, the maximum stress occurred at the angle between the inner and outer cells. Figure 16c reveals the deformation mode by extracting continuous honeycomb walls. It is shown that the vertical force exerting on the top node was gradually divided into a parallel pressure and vertical torque. Consequently, the auxetic structure with a lower angle pushed the inclined beam, which suffered a larger vertical compression and became deflected, making the structure fold inward and ultimately leading to an overall height reduction [37]. This mechanism explains why T1 achieved the highest compressive strain while T3 obtained the lowest strain. 

When the honeycomb angle increased, the honeycomb wall acted similarly to a straight spoke, which can bear more vertical force and maintain stability. Figure 16d further shows the impact of the cellular angle on the maximum stress and strain values. The maximum strain of the structure was controlled within 2%. The maximum stress corresponding to T1 (*α* = 90°) reached 35 Mpa, and when the angle increased to T3 (*α* = 145°), the maximum stress decreased to 12 MPa with a reduction ratio of 66%, which plays an important role in preventing fatigue damage of the honeycomb [43]. However, as an NPT support structure, it needs to undergo certain deformation under load to absorb the ground recoil energy. An incline angle is helpful to increase the flexibility of the honeycomb structure. Within the base elastic range, a limited incline angle is necessary to consider.

**Figure 17:**
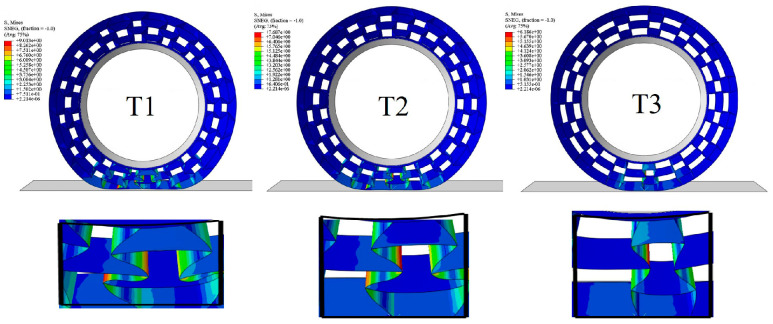
Support structure with material parameters of TPU and vertical load of 1.5 kN.

For comparison, the mechanical properties of TPU were assigned to the same model, using material parameters cited from [44]. The vertical load was reduced to 1.5 kN, and the analysis results are shown in Figure 17. While the load was reduced by 70%, the structure still had a greater deformation, which preliminarily reflects the insufficient bearing capacity of TPU as the material for the NPT support structure.

**Figure 18:**
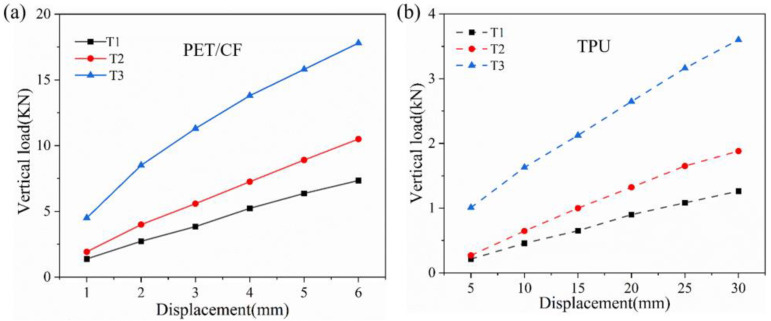
Load–displacement curves of NPT with support structure based on two different materials, (**a**) PET/CF and (**b**)TPU.

In order to further study the loading capacity of the NPT support structure, the boundary conditions were changed to make the rigid hub center fixed, and the lifting displacement was applied to the ground. The reaction force at the ground position of the tire at this time was equivalent to the vertical load that caused the deformation, and the continuous force–displacement data were obtained using this method. As shown in Figure 18, the bearing capacity of the support structure can be controlled in a wide range by changing the honeycomb angle. The TPU-based structure exhibited a similar pattern. Although a larger deformation of 30 mm (nearly 5% of the total size) was observed, the acceptable load was still less than 4 kN. As a support structure, the PET/CF honeycomb exhibited a good load-bearing capacity and maintained a stable operation under larger loads.

Vertical stiffness is an important performance for the operating stability of an NPT and is defined in Formula (1) [45], where *F* is the vertical load and *δy* is the directional deformation, which is equivalent to the lifting displacement of the ground.

(1) $$K=\frac{F}{\delta_{y}}$$

**Figure 19:**
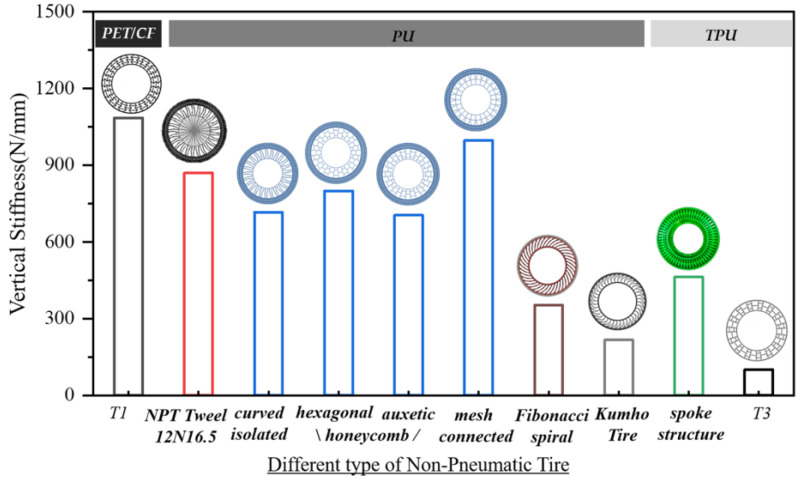
Vertical stiffness of different types of NPT based on different polymeric materials. The PET/CF material was used for the auxetic structure in this study, and the rest of the material and NPT structures were considered as controls. NPT Tweel 12N16.5 [46], curved isolated, hexagonal honeycomb, auxetic honeycomb, mesh connected [4], Fibonacci spiral [13] and Kumho tire [45] used PU material. Spoke structure [26] and T3 in this study used TPU.

The deformation of NPTs is approximately linearly related to the load. In research, the vertical stiffness of a tire is generally regarded as a fixed value. To compare the tire stiffness with that of a real tire or other prototype support structures, relevant literature about the NPT with different infill structures were listed in Figure 19, and the vertical stiffness in the current work was compared with those reference values [4,13,26,45,46]. Most of the studies were carried out through numerical analysis except for the *Tweel 12N16.5* which was studied as a real tire using an experimental test. It can be observed that the value of vertical stiffness in the PET/CF-based NPT structure is slightly higher than that of the real tire and other proposed PU-based types, indicating an improvement in the load-bearing capacity of the PET/CF auxetic support structure under a small deformation. On the other hand, although thermoplastic polyurethane (TPU) can be fabricated into filaments and printed into a prototype, the corresponding support structures show limited vertical stiffness, which restrict their future development in NPT applications. Therefore, the comparison results combined with the material and structural findings demonstrate that the PET/CF honeycomb structure possesses a superior load-carrying capacity when used as a support structure for an NPT in contrast with currently available types or materials. 

## 5. Conclusions

In this paper, PET/CF, a kind of carbon fiber-reinforced thermoplastic material, was proposed to be 3D-printed into a prototype for an NPT support structure. Through experimental testing and finite element analysis, the compressive properties of the honeycomb structure were evaluated and the influence of geometric parameters on the structural performance were assessed. Furthermore, the vertical stiffness of the NPT support structure based on PET/CF was evaluated under a static load and this was compared with TPU material under the same working conditions. The following conclusions can be listed as follows.

(1) The PET/CF proved to possess excellent mechanical properties and good 3D printability. The PET/CF auxetic honeycomb applied as a support structure in an NPT represents a convenient option from geometric design to prototype fabrication.
(2) The quasi-static compression experiment showed that the deformation mode and force displacement results were in good agreement with the actual data, which could be used to predict the compression performance of the structure. In the basic mechanics simulation, the compression performance and natural frequency of the honeycomb structure were positively correlated with the element angle and the wall thickness. The high support force initially showed the bearing potential of the structure.
(3) The support structure of the PET/CF-based NPT had both a high modulus of material and the flexibility of a honeycomb. By changing the honeycomb angle, the deformation of the structure can be regulated on a large scale. High vertical stiffness ensures the stability of the tire. In comparison, it also exhibits superior load-carrying capacity to the TPU-based NPT counterparts.

Therefore, the current work proposes a 3D-printed PET/CF honeycomb prototype with good load-bearing capacity, which is suitable as an NPT supporting structure. The deformation mechanism and the vertical loading performance could be modulated by altering the angle of the cellular unit. Since the vertical stiffness of the support structure is higher than that of a real tire, it provides variable space for the tread, and can be considered to combine with elastomer polymer to form a complete NPT. In addition, a lateral and longitudinal stiffness analysis could also be considered for the assessment of NPTs.

## Figures and Tables

**Table 1:** Mechanical properties of the 3D-printed PET/CF samples from experimental data.

Sample	1	2	3	Mean	SD
Tensile modulus (MPa)	3473.1	3391.6	3462.2	3442.3	44.2
Tensile strength (MPa)	82.5	82.2	76.9	80.5	3.2
Flexural modulus (MPa)	6840	6803.5	7130.5	6924.7	179.2
Flexural strength (MPa)	120.3	119.5	118.4	119.4	1.0

## Data Availability

Data are contained within the article.
